# Online Hearing Voices Peer Support Groups: Assessing Feasibility and Acceptability Within UK National Health Service Settings

**DOI:** 10.1093/schizbullopen/sgaf002

**Published:** 2025-02-04

**Authors:** Alison Branitsky, Samantha Bowe, Anthony P Morrison, Eleanor Longden, Sandra Bucci, Lee D Mulligan, Filippo Varese

**Affiliations:** Division of Psychology and Mental Health, School of Health Sciences, Faculty of Biology, Medicine and Health, Manchester Academic Health Sciences Centre, The University of Manchester, Manchester, M13 9PL, United Kingdom; Psychosis Research Unit, Greater Manchester Mental Health NHS Foundation Trust, Manchester, M25 3BL, United Kingdom; Complex Trauma and Resilience Research Unit, Greater Manchester Mental Health NHS Foundation Trust, Manchester, M13 9PL, United Kingdom; Psychosis Research Unit, Greater Manchester Mental Health NHS Foundation Trust, Manchester, M25 3BL, United Kingdom; Division of Psychology and Mental Health, School of Health Sciences, Faculty of Biology, Medicine and Health, Manchester Academic Health Sciences Centre, The University of Manchester, Manchester, M13 9PL, United Kingdom; Psychosis Research Unit, Greater Manchester Mental Health NHS Foundation Trust, Manchester, M25 3BL, United Kingdom; Division of Psychology and Mental Health, School of Health Sciences, Faculty of Biology, Medicine and Health, Manchester Academic Health Sciences Centre, The University of Manchester, Manchester, M13 9PL, United Kingdom; Psychosis Research Unit, Greater Manchester Mental Health NHS Foundation Trust, Manchester, M25 3BL, United Kingdom; Complex Trauma and Resilience Research Unit, Greater Manchester Mental Health NHS Foundation Trust, Manchester, M13 9PL, United Kingdom; Division of Psychology and Mental Health, School of Health Sciences, Faculty of Biology, Medicine and Health, Manchester Academic Health Sciences Centre, The University of Manchester, Manchester, M13 9PL, United Kingdom; Complex Trauma and Resilience Research Unit, Greater Manchester Mental Health NHS Foundation Trust, Manchester, M13 9PL, United Kingdom; Division of Psychology and Mental Health, School of Health Sciences, Faculty of Biology, Medicine and Health, Manchester Academic Health Sciences Centre, The University of Manchester, Manchester, M13 9PL, United Kingdom; Division of Psychology and Mental Health, School of Health Sciences, Faculty of Biology, Medicine and Health, Manchester Academic Health Sciences Centre, The University of Manchester, Manchester, M13 9PL, United Kingdom; Complex Trauma and Resilience Research Unit, Greater Manchester Mental Health NHS Foundation Trust, Manchester, M13 9PL, United Kingdom

**Keywords:** hearing voices network, peer support, voice hearing, online support, feasibility study

## Abstract

**Background:**

User-led hearing voices groups (HVGs) have existed for the past 40 years, but little research has been conducted into if and how this approach can be implemented in statutory mental health settings, and whether they can be delivered effectively when held online. The aim of this study was to conduct a feasibility and acceptability trial of an online HVG for the UK National Health Service (NHS) users who hear voices, to inform a future larger-scale trial.

**Methods:**

A mixed-methods, nonrandomized feasibility study of an online HVG was conducted with 9 participants. Participants attended weekly online meetings for 6 months and completed measures of social connectedness, voice hearing, personal recovery, as well as semi-structured interviews, at baseline and postintervention (26-weeks). Primary outcomes were qualitative and quantitative assessments of feasibility and acceptability.

**Results:**

Thirty-eight participants were referred to the trial, 9 of whom were recruited (100% of the target sample). The trial had high retention (100%) and engagement (mean = 13.2 groups attended). Participants reported positive features of attending this digital intervention, with 85% reporting it was helpful to meet other voice hearers, that the group helped them make sense of voice hearing experiences, that they received positive messages about recovery, and that the group represented a form of support they could not get elsewhere.

**Discussion:**

The findings indicate it is feasible and acceptable to run an online HVG within an NHS setting. A larger trial is needed to further investigate the utility, efficacy, and cost-effectiveness of running online HVGs in the NHS.

**Trial Registration:**

ISRCTN11873550.

## Introduction

The presence of hearing voices groups (HVGs) has increased across the world over the past 40 years and HVGs are now one of the most widespread and influential user-led initiatives in mental health.^1^ Establishing and running HVGs is one of the main prerogatives of the hearing voices movement (HVM), a grassroots initiative of voice hearers and their allies which promotes a person-centered, meaning-based, and recovery-oriented approach to voice hearing and other unshared perceptual experiences.^2,3^ In this respect, HVGs have a longstanding tradition in third-sector and voluntary settings, with over 100 operating in the United Kingdom^4^ and comparable groups currently running in 25 countries across the world.^5^ However, the HVM takes a different ideological and epistemic position to that of traditional mental health research and service delivery^3^: HVM is strongly aligned with the sociopolitical psychiatric survivors and consumer/survivor/ex-patient movements and accordingly, values individual testimony over predefined clinical outcomes.^1^ As such, controlled investigations into these groups have only recently started to take place. Likewise, while HVGs have begun to be run in the United Kingdom as part of National Health Service (NHS) services, to date, there has been neither a critical evaluation of how HVGs should be run in the context of clinical services nor have relevant clinical or recovery-oriented outcomes been reported.

Existing qualitative research into community-based HVGs indicates that groups are distinct from many other existing forms of mental health support in terms of both their style of interaction and content of meetings,^6^ with emphasis placed on self-determination, egalitarian collaboration, open conversation, respect for all explanations for voice hearing, and the fostering of genuine, mutual relationships between group members.^7^ Accordingly, a systematic review by Corentin et al^8^ notes that participants report numerous benefits from group participation, including increased social connectedness and decreased isolation, enhanced understanding and coping with voices, and bolstered self-confidence and hope for the future.

Kalofonos et al^9^ conducted the first controlled study of HVGs adapted to take place within a large public healthcare system in the United States. Twenty-nine individuals took part in a 16-week HVG which, due to the COVID-19 pandemic, met online. Results indicated that group participation resulted in a significant reduction in voice-related distress, a reduction in beliefs about voice omnipotence and malevolence, and an increase in beliefs about voice benevolence. McManus et al^10^ likewise piloted a HVG on an inpatient ward in the UK NHS and found that participants valued having an opportunity to speak to others with similar experiences and learn new coping skills. However, while the findings suggested that it was possible and worthwhile to adapt HVGs to be delivered within the NHS, clinical outcome data were not reported.

Prior to the COVID-19 pandemic, only 2 HVGs worldwide operated remotely via Zoom.^11^ National lockdowns forced many groups online; while some of these groups have now transitioned back to face-to-face, others have remained online, and others still were founded with the explicit intention of operating solely via videoconference. Online groups offer many opportunities: the ability to join from one’s home may increase accessibility, and the disinhibition experienced by many online may reduce power differentials and may promote self-disclosure.^12^ But they also come with challenges, particularly to group cohesion, which is an important consideration in the context of a mutual support group. Weinberg^13^ describes numerous challenges to establishing cohesive groups online, including technical glitches, the unnaturally linear flow of dialogue, the potential for inhibited sharing, and the ease of disengagement online. Absence of eye contact,^12^ limited nonverbal cues,^14^ and the ability for participants to turn their cameras off^15^ may all present additional barriers to establishing group cohesion. There may also be specific challenges to engaging voice hearers online; Watson et al^16^ found that a sizable minority (37%) of individuals diagnosed with psychosis declined remote therapy because of the impact of voices or distressing beliefs. Hearing voices group facilitators have, however, indicated that it is possible to foster group cohesion online and that voice-related barriers to engaging in online HVGs were not as prevalent as feared.^17^ Ultimately, facilitators asserted that these groups may represent a crucial option for individuals who are unable or unwilling to attend a group face-to-face.

Given the current scarcity of the provision of psychological interventions in the NHS, there is a need for resource-efficient forms of support, such as online support groups, to be investigated. Indeed, the National Clinical Audit for Psychosis^18^ estimates that less than half of the individuals in early interventions for psychosis (EIS) services had accessed at least one session of cognitive behavioral therapy for psychosis (CBTp), the therapeutic intervention recommended by the National Institute of Health and Care Excellence (NICE) for individuals distressed by their voices. CBTp may likewise be unavailable to voice hearers without a psychosis diagnosis,^19^ may not adequately address distressing voices specifically,^20,21^ and may not always be available in clinical services due to implementation and funding challenges.^22^ As an existing community resource, HVGs present a promising option to provide support for distressed voice hearers. Furthermore, the NHS Long Term Plan^23^ has identified the more widespread provision of digital forms of mental health support as one of its priorities. Accordingly, the aim of this study was to conduct the first mixed-methods feasibility trial of an online HVG conducted within a UK NHS setting. The primary study objective was to assess the feasibility, acceptability, and safety of delivering an online HVG in the NHS to inform a future definitive trial and to develop and refine the processes for delivering the group in the NHS.

## Methods

### Design

We conducted a nonrandomized feasibility trial with a 26-week intervention period and preintervention and postintervention assessments with a nested qualitative study.

### Participants

Previous research^24^ and patient and public involvement and engagement (PPIE) consultation deemed that at least 6 participants were necessary to foster adequate group cohesion and diversity of experience, while more than 10 participants could disrupt cohesion online. As such, a target sample size of 6–10 participants was deemed appropriate.

Eligible participants were aged ≥18 years, were currently living in the United Kingdom, had heard voices for ≥6 months, were willing to engage in group support, had consistent access to the internet and the ability to use videoconferencing platforms, and were able to provide informed consent. Participants did not have to be in current receipt of NHS mental health services to be eligible. Participants were excluded if they were at immediate risk of harm to self or others or if they were non-English speaking. A total of 9 participants took part in this study. Six eligible participants were referred from EIS or community mental health teams (CMHTs) within Greater Manchester Mental Health NHS Foundation Trust (GMMH). The final 3 eligible participants self-referred but were all in receipt of CMHT support from services outside the host center.

### Assessments

Written informed consent was obtained prior to gathering research data. The University of Manchester acted as the study sponsor. Participants were recruited from GMMH NHS Foundation Trust, voluntary/third-sector mental health services, and from social media. The study was prospectively registered (ISRCTN 11873550) and was approved by the NHS West Midlands—Black Country Research Ethics Committee (23/WM/0045).

The full protocol of study procedures is provided elsewhere.^24^ In brief, eligible participants completed study assessments and individual qualitative interviews at baseline and postintervention (26-weeks). Additional assessments of group cohesion were completed at weeks 4, 12, and postintervention. Interviews took place remotely via Zoom. Assessment measures were self-directed and were completed remotely via Qualtrics, with the option for researcher support if requested. Participants were compensated £20 for the baseline and postintervention interview and assessments.

### Online Hearing Voices Group

All participants were invited to attend the online HVG, which met on Zoom for one 90-min session per week over the course of 26 weeks. Participants were allowed to attend the group with a carer if desired. The HVG was delivered by a trained HVG peer facilitator (A.B.) and a clinical psychologist (S. Bowe or L.D.M.). In accordance with the ethos of the HVM, the group was unstructured and had no set agenda. Rather, participants were encouraged to raise topics that were relevant to them and were asked to provide reflections on the contributions of other members. Topics of conversation were wide-reaching, but largely focused on coping with voices; understanding their potential origin and meaning; exploring the relationship between voice hearing, mental health, and identity; and processing adverse life experiences which may be implicated in voice hearing. Previously identified recommendations for fostering group cohesion^17^ were adhered to in order to optimize running the HVG online.

### Outcomes

The primary outcome was the feasibility and acceptability of delivering an HVG online within a UK NHS context. In line with CONSORT recommendations,^25^ feasibility was assessed by: the number of eligible participants consenting, total number of participants recruited, completeness of outcome measures, group attendance rates, study drop-out rate, reason for withdrawal, suitability of outcome measures, suitability of trial procedures, and suitability of the intervention. Suitability of outcome measures was assessed via completion rates and qualitative feedback; suitability of trial procedures and intervention were assessed via retention and withdrawal rates and reason and through qualitative feedback. Acceptability was assessed via the HVG Survey (described below) and through qualitative interviews which will be reported elsewhere.

As HVGs are not structured interventions, do not advocate for specific outcomes, and understand benefits as subjective,^7,26^ traditional clinical assessment measures were deemed inappropriate. Secondary outcome measures therefore focused on central domains and proposed mechanisms of change in HVGs as identified by Hornstein et al^6^ and Corentin et al^8^: social support, voice hearing, and personal recovery. A summary of outcome measures is presented in Table 1.

**Table 1.** Summary and Description of Outcome Measures

Scale	Score range and interpretation
Social comparison scale^27^	Range: 11–111 Higher scores indicate more favorable social comparison
Social connectedness scale—revised (SCS-R)^28^	Range: 20–120 Higher scores indicate higher social connectedness
UCLA loneliness scale^29^	Range: 20–80 Higher scores indicate more isolation and loneliness
Approve questionnaires—social^30^	Three subscales of assertive responding to others, aggressive relating to others, and submissive relating to others Range: 0–50 per subscale Higher scores indicate more assertive, aggressive, and submissive responding, respectively
Approve questionnaires—voices^30^	Three subscales of assertive responding to voices, aggressive relating to voices, and submissive relating to voices Range: 0–50 per subscale Higher scores indicate more assertive, aggressive, and submissive responding, respectively
Voices impact scale (VIS)^31^	Range: 0–240 Higher scores indicate a more distressing emotional impact of voices
Voice acceptance or action scale-12 (VAAS-12)^32^	Range: 12–60 Higher scores indicate more acceptance of voices
Personal beliefs about experiences questionnaire (PBEQ)^33^	Range: 13–52 Higher scores indicate more negative views about voice hearing
Questionnaire about the process of recovery (QPR-15)^34^	Range: 15–75 Higher scores indicate higher perception of recovery
Group cohesiveness scale (GCS)^35^	Range: 7–35 Higher scores indicate more perceived group cohesion
Therapeutic factors inventory—short form (TFI-S)^36^	Four subscales of instillation of hope, secure emotional expression, awareness of relational impact, and social learning Range: 6–42 (instillation of hope) 7–49 (secure emotional expression) 6–42 (awareness of relational impact) 4–28 (social learning) Higher scores indicate higher endorsement of each subscale
Hearing voices group survey (HVGS)^37^	Three subscales of experiences in the group, impact of membership on life outside the group, and the effect of the group on emotional well-being Scores are reported per-item

To assess safety, adverse events (AEs) were monitored and recorded using standardized operating procedures consistent with UK Health Research Authority and local research and development policies of participating NHS organizations, including participant self-report and screening of electronic medical records for participants who were recruited from GMMH. Electronic medical records were screened by A.B., in consultation with S. Bowe and F.V. All AEs were reviewed for relatedness to study procedures and serious adverse events (SAEs) were reviewed by 2 clinically qualified senior researchers and reported to the study sponsor for continued monitoring.

### Statistical Analysis

As a feasibility trial, the study was not powered to detect clinical efficacy; therefore, no inferential tests were conducted. Analyses followed a prespecified plan (available at www.isrctn.com/ISRCTN11873550). In line with the CONSORT statement for pilot and feasibility trials, recruitment and retention data were summarized using descriptive statistics.^25^ Outcome data were summarized as mean (SD) for continuous variables and frequencies/percentages for categorical variables. Pre- and postintervention questionnaire data were further examined by computing change scores and associated 95% confidence intervals (CIs). Missing data were imputed in instances where ≤20% of the data were missing in a single scale. In instances where >20 % of the data were missing, the outcome was marked as missing.

## Results

**Figure 1.**
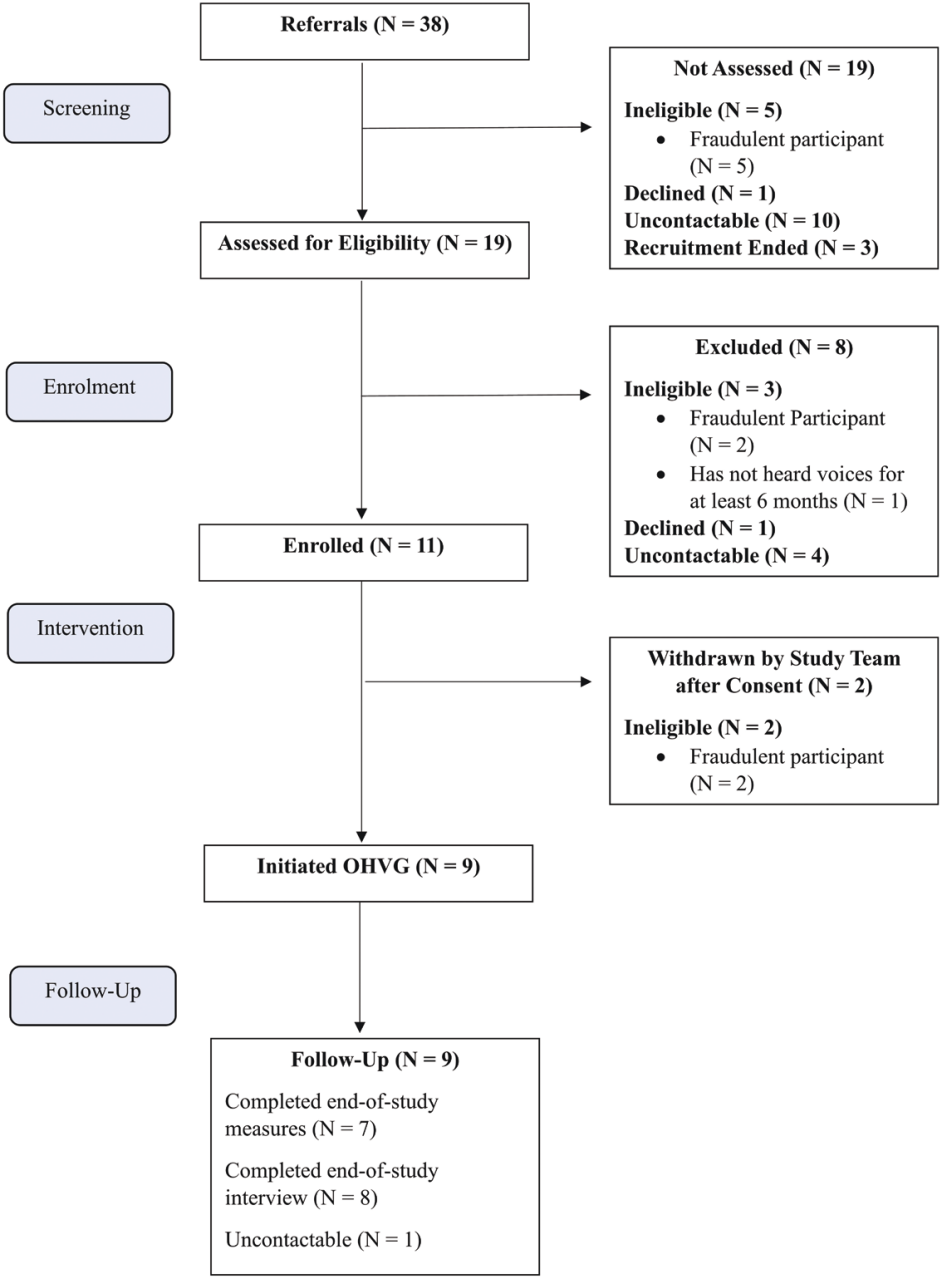
CONSORT Diagram for Flow of Participants. Abbreviation: OHVG = online hearing voices group

Recruitment for the study took place between April 17, 2023 and October 5, 2023. Eligibility and baseline assessments took place within 2 weeks of referral. The online HVG commenced on September 18, 2023. One participant was recruited after the group had begun. Follow-up assessments were conducted between March 27, 2024 and May 7, 2024. Baseline characteristics are summarized in Table 2 and a CONSORT diagram depicting participant flow is presented in Figure 1.

**Table 2.** Baseline Characteristics of the Sample

	Total sample (*N* = 9)
Age (years)—mean (SD)	34.6 (15.0) (range: 18–58)
Gender—*n* (%)	
Female	4 (44.4%)
Male	5 (55.6%)
Ethnicity—*n* (%)	
White Caucasian	7 (77.7%)
Asian	1 (11.1%)
Mixed-race	1 (11.1%)
Marital status—*n* (%)	
Single	6 (66.7%)
Married/living with partner	1 (11.1%)
In a relationship but not living together	1 (11.1%)
Divorced	1 (11.1%)
Employment status—*n* (%)	
Unemployed	5 (55.6%)
Student	2 (22.2%)
Exempt through disability	2 (22.2%)
Highest educational level—*n* (%)	
Secondary education	5 (55.6%)
Tertiary/further education	3 (33.3%)
Other general education	1 (11.1%)
Voice hearing duration (years)—mean (SD)	11.7 (15.5) (range: 1–44)
Heard voices continuously since onset—*n* (%)	
Yes	9 (100%)
No	0 (0%)
Self-reported diagnoses—*n* (%)	
Schizophrenia	1 (11.1%)
Schizoaffective	1 (11.1%)
Psychosis	2 (22.2%)
Bipolar disorder	1 (11.1%)
Depression	3 (33.3%)
Anxiety disorder	3 (33.3%)
Complex post-traumatic stress disorder	1 (11.1%)
Post-traumatic stress disorder	1 (11.1%)
Autism spectrum disorder	3 (33.3%)
None	2 (22.2%)
Type of NHS mental health service	
Early detection and intervention	1 (11.1%)
Early intervention in psychosis	5 (55.5%)
Community mental health team	3 (33.3%)
Past psychiatric hospitalization—*n* (%)	
Yes, multiple times	4 (44.4%)
Yes, one time	1 (11.1%)
No	4 (44.4%)
Current psychiatric medication use—*n* (%)	
Yes	9 (100%)
No	0 (0%)
Past therapy for mental health—*n* (%)	
Yes	8 (88.9%)
No	1 (11.1%)

### Feasibility Outcomes

Recruitment rates were 100% of the target sample. The conversion rate was approximately 4:1, with 10 referred individuals being ineligible (26.3%), 14 uncontactable (28.9%), and 2 declining to participate (5.2%). Most referrals came from social media (50%); however, none of these referrals were ultimately eligible. Following eligibility assessments, all social media referrals were deemed to be from fraudulent participants (eg, all referrals were received in rapid succession; email addresses and content followed a standard, repetitive format; individuals refused to turn on their cameras during eligibility screenings and audio was unclear; individuals provided fabricated or nonsensical phone numbers, addresses and GPs; individuals were unable to answer questions about their voice hearing experiences).^38,39^ Two participants who were screened for eligibility and consented to the study were withdrawn and signposted to a community-based HVG prior to the group commencing after having been discovered to be fraudulent participants. The most frequently referred service was EIS teams (28.9%), followed by CMHTs (7.9%) and self-referrals from individuals in CMHTs in NHS Trusts outside GMMH (7.9%). Referral and recruitment rates by service type are displayed in Table 3.

**Table 3.** Referral and Recruitment of Participants By Service Type

	Referred	Eligible	Consented
Early detection and intervention (EDIT)	1	1	1
EIS	11	5	4
CMHT	3	2	1
Third-party/voluntary organization	0	0	0
Self-referral (under non-GMMH mental health team)^a^	4	3	3
Social media	19	4	2^b^

^a^One participant was open to EIS and 2 were open to CMHTs.

^b^Withdrawn prior to group commencing as it was determined they were fraudulent participants and therefore became ineligible.

Nine participants were retained in the group by the end of the intervention (100%), with seven (77.8%) completing the postintervention measures and 8 (88.9%) completing the postintervention interview. A total of 23 group sessions were held between September 18, 2023 and March 25, 2024. The range of attendance across participants was 5–23 groups (mean = 13.2 groups, SD = 5.9, and median = 12 groups).

Due to the small sample size, formal analyses of secondary outcome data were not conducted. Descriptive statistics, adjusted mean differences, standard errors, and 95% CI of secondary outcomes pre- and postintervention are presented in Table 4. Summary statistics of group cohesion outcomes taken at 4 weeks, 12 weeks, and 26 weeks are presented in Table 5. In brief, no significant changes were found across the domains of social connectedness, voice hearing, or feelings of personal recovery between pre- and postintervention. Furthermore, no changes in group cohesion were observed.

**Table 4.** Descriptive Statistics and Mean Change for Secondary Outcome Measures at Pre- and Postintervention

	Preintervention (SD) (*n* = 8)	Postintervention mean (SD) (*n* = 7)	Adjusted mean difference (SE)	95% CI
Social comparison scale	46.4 (19.0)	57.0 (16.3)	9.3 (5.5)	(−22.7, 4.2)
SCS-R	64.8 (17.5)	65.7 (11.8)	−1.4 (5.0)	(−10.7, 13.6)
UCLA loneliness scale	53.4 (11.8)	50.4 (7.0)	−0.1 (2.1)	(−5.1, 5.4)
Approve social—assertive responding	28.5 (9.6)	29.2 (11.8)	0.7 (2.4)	(−6.8, 5.4)
Approve social—aggressive relating	20.7 (15.8)	27.5 (17.7)	6.8 (3.7)	(−16.3, 2.6)
Approve social—submissive relating	20.7 (8.5)	22.5 (11.9)	1.8 (4.4)	(−13.2, 9.5)
Approve voices—assertive responding	23.1 (9.7)	29.2 (12.9)	4.5 (4.3)	(−15.6, 6.6)
Approve voices—aggressive relating	31.0 (10.9)	30.6 (16.3)	−2.3 (4.8)	(−13.2, 8.7)
Approve voices—submissive relating	33.4 (11.7)	31.3 (7.7)	−0.3 (3.4)	(−8.7, 8.1)
VIS	176.9 (42.4)	151.1 (33.4)	−21.6 (16.0)	(−17.7, 60.8)
VAAS-12	33.6 (6.2)	37.0 (5.3)	2.3 (2.4)	(−8.3, 3.7)
PBEQ	37.5 (7.1)	32.6 (4.3)	−3.0 (2.5)	(−3.0, 9.0)
QPR	41.6 (12.2)	46.3 (7.8)	2.6 (2.4)	(−8.3, 3.2)

Abbreviations: PBEQ, personal beliefs about experiences questionnaire^33^; QPR, questionnaire about the process of recovery^34^; SCS-R, social connectedness scale—revised^28^; VAAS-12, voice acceptance and action scale—12^32^; VIS, voices impact scale^31^.

**Table 5.** Descriptive Statistics for Group Cohesion Measures at 4 weeks, 12 weeks, and 26 weeks

	4-week mean (SD) (*n* = 6)	12-week mean (SD) (*n* = 6)	26-week mean (SD) (*n* = 7)	4-week to 12-week adjusted mean difference (SE)	95% CI	4-week to 26-week adjusted mean difference (SE)	95% CI
GCS	28.2 (3.5)	26.8 (3.1)	27.3 (5.4)	−0.2 (0.5)	(−1.2, 1.6)	−1.7 (1.9)	(−3.3, 6.7)
TFI-S—instillation of hope	30.7 (7.5)	30.2 (7.4)	31.4 (5.2)	−0.8 (2.1)	(−4.9, 6.5)	0.3 (1.9)	(−5.1, 4.4)
TFI-S—secure emotional expression	33.3 (9.6)	33.7 (7.8)	34.9 (7.7)	1.4 (2.0)	(−6.9, 4.1)	0.7 (2.1)	(−6.0, 4.7)
TFI-S—awareness of relational impact	29.2 (5.8)	30.3 (6.9)	28.4 (5.0)	1.4 (0.9)	(−4.0, 1.2)	−0.7 (1.2)	(−2.4, 3.8)
TFI-S—social learning	17.2 (3.4)	19.8 (4.4)	19.7 (5.2)	3.2 (2.5)	(−10.2, 3.8)	2.0 (2.4)	(−8.2, 4.2)

Abbreviations: GCS, group cohesion scale^35^; TFI-S, therapeutic factors inventory—short form^36^.

### Acceptability Outcomes

**Figure 2.**
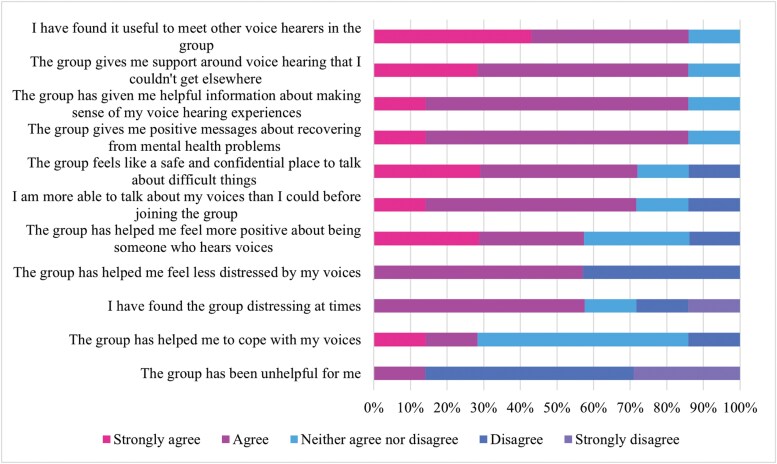
HVGS Outcomes on Experiences in the Group. Abbreviation: HVGS, hearing voices group survey

Participants reported several positive experiences in, and impacts of, taking part in the online HVG. When considering experiences in the group, the most strongly endorsed items were: (a) it was useful to meet other voice hearers in the group (85% at least agree); (b) the group provided support around voice hearing that members could not get elsewhere (85%); (c) the group provided useful information for making sense of voice hearing (85%); and (d) the group provides positive messages about recovering from mental health problems (85%). Furthermore, most participants stated that the group was a safe place to talk about difficult things (72%), they felt more able to talk about their voices than they were prior to joining the group (72%), they felt more positive about being someone who hears voices (58%), and that the group has helped them feel less distressed by their voices (58%). Notably, while the majority (58%) of participants reported that they found the group distressing at times, most participants nevertheless endorsed positive experiences both in and beyond the group. Frequency outcomes on participants’ experiences in the group are reported in Figure 2.

**Figure 3.**
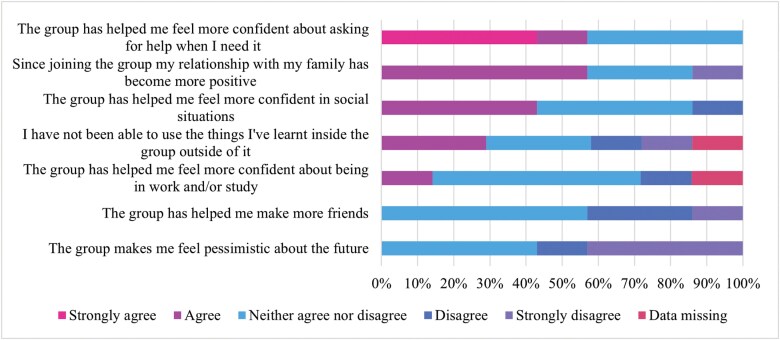
HVGS Outcomes on Impact of Online HVG Participation on Life Outside the Group. Abbreviation: HVGS, hearing voices group survey

Participants likewise reported numerous benefits of online HVG participation in life outside the group. Most participants reported that the group helped them feel more confident asking for help when they needed it (58%) and that since joining the group, their relationship with their family has become more positive (58%). Likewise, most participants disagreed that the group made them feel more pessimistic about the future (58%). A sizeable minority of participants also stated that the group helped them feel more confident in social situations (43%). An even split of participants reported that they have been able to use things they have learned within the group outside of it (29% agree, 29% neither, and 29% disagree). Frequency outcomes on the impact of online HVG participation on life outside the group are reported in Figure 3.

**Figure 4.**
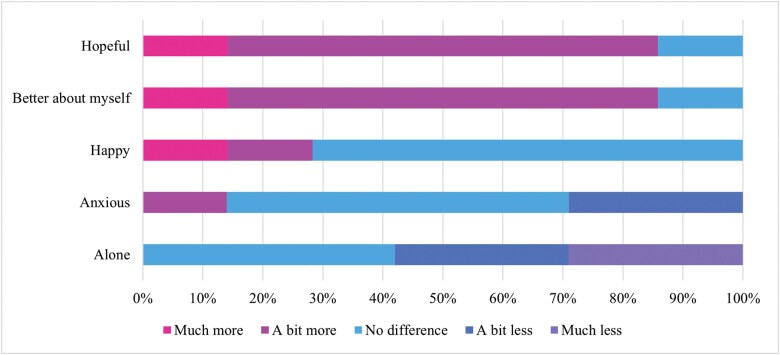
HVGS Outcomes on the Effect of the Group on Emotional Well-Being. Abbreviation: HVGS, hearing voices group survey

The emotional impact of the group was likewise noted by participants, with 85% of participants reporting they felt better about themselves, 85% reporting feeling more hopeful, and 58% reporting feeling less alone. Frequency outcomes on the effect of the group on emotional well-being are reported in Figure 4.

### Safety Outcomes

The incidence of AE is reported in Table 6, all of which were classified as unrelated to trial procedures or the group. We recorded 4 AEs in 1 participant and 6 SAEs in 4 participants. One SAE (suicidal ideation with behavioral component) occurred after consent, but prior to the group commencing.

**Table 6.** Incidence of Adverse Events

*N* = 9	(*N*/%)
Adverse events	
Participants with an AE	1 (11.1%)
Number of AEs	4
Types of AE	
Self-harm	1
Increase in distress requiring change in care	3
Serious adverse events	
Participants with an SAE	4 (44.4%)
Number of SAEs	6
Types of SAE	
Voluntary psychiatric admission	2
Suicidal ideation with behavioral component	2
Otherwise medically significant (seizure)	1
Otherwise medically significant (ACL tear)	1
AEs/SAEs related to study procedures	
Participants with a related AE/SAE	0 (0%)
Number of AEs/SAEs related to study procedures	0

## Discussion

The current study demonstrates that it is possible to recruit, retain, and engage individuals who hear voices in an online HVG run in an NHS context, providing indications of feasibility. Participants likewise reported numerous positive experiences in, and impacts of the online HVG, including that the group offered a unique form of support that could not be found elsewhere. Furthermore, there were no emergent safety concerns resulting from group participation. These are encouraging findings, given both the ideological differences between the HVM and NHS services and the challenges with running cohesive groups online, which together could have represented a barrier to effectively facilitating such groups in a public healthcare system. Given that this was a feasibility trial with a small sample size, future studies are needed to draw conclusions about the potential clinical efficacy and cost-effectiveness of groups.

The study had low attrition (0%) and high levels of engagement, with 7 (77.8%) participants completing the full postintervention assessment battery. Participants attended a mean of 13 group sessions, which is comparable to findings investigating HVGs in other public healthcare settings.^9^ Furthermore, Longden et al^37^ found that group satisfaction was comparable between individuals who had attended HVGs for between 1 and 6 months and individuals who had attended for over 6 months, indicating that any impact may be experienced early in group participation.

In line with HVM ethos, a comprehensive clinical assessment of voice hearing (eg, using the Positive and Negative Syndrome Scale^40^) was not conducted. Therefore, it is not possible to extrapolate whether the current sample is representative of voice hearing population within secondary mental health services. While all participants were currently in receipt of NHS mental health care, the average duration of voice hearing in the current study was less than that reported in studies with a similar population.^41,42^ This is likely since the majority (66.7%) of participants in the current study were open to early detection and intervention teams (EDIT) or EIS teams. Furthermore, most participants in this study had received psychological therapy (88.9%), which is significantly higher than the national rate of CBTp uptake.^18^ Further research is therefore warranted to discern whether those who have had access to psychological support are more willing to participate in peer interventions.

The acceptability outcomes (HVGS) indicate that the group was well-liked by participants and yielded a variety of benefits. Notably, 2 of the most endorsed items, “*I have found it useful to meet other voice hearers in the group*” and “*the group has given me support around voice hearing that I couldn’t get elsewhere*” speak to the distinct value of peer support which may provide unique benefits beyond what is currently offered by mental health services. The other acceptability findings are largely in line with previous research, namely, that having the opportunity to meet other voice hearers, reframe voice hearing experiences, and speak openly about difficult topics may help facilitate self-acceptance, and increase hope, connection, and confidence.^6,8,9^ Importantly, while 58% of participants reported that they found the group distressing at times, they nevertheless reported benefits to group participation. This is again in line with previous research^37^ which stipulates that benefits can be attained despite the inherent difficulties that come with discussing voice hearing and associated experiences. Indeed, in this study, distress likely arose from fears around social rejection, backlash from voices following the disclosure of voice content, and discussions of social adversity; this is explored more fully in the qualitative findings.

Additional work is now needed to understand the potential clinical effects of online HVG participation with a larger sample. The secondary outcome measures suggest no change in the domains of social connectedness, impact of voice hearing, or personal recovery; however, given previous qualitative findings,^6–8,26^ it is worth continuing to investigate these domains in a larger powered sample. Given that HVGs are intentionally broad in content and focus significant attention on understanding voice meaning, origin, and relationship to the hearer, future studies may benefit from including a wider spectrum of voice hearing outcomes such as the Revised Beliefs about Voices Questionnaire (BAVQ-R),^43^ Perth Voice Content Questionnaire (PVCQ),^44^ and the Yale Control Over Perceptual Experiences Scales (COPE),^45^ as well as additional measures pertaining to sense of self (eg, Internalised Stigma of Mental Illness scale [ISMI],^46^ Self-Esteem Rating Scale—Short Form [SERS-S],^47^ and Internalised Shame Scale [ISS]^48^) to capture potential effects that may not have been detected in the present study. While the present study was not adequately powered to detect changes in measures pertaining to social connectedness, voice hearing, and perceptions of personal recovery, it is nevertheless worth continuing to explore these outcomes in the future as both theoretical and qualitative literature attests to their importance.^6–8,26^ It is worth noting that previous research has remained critical of using clinical outcome measures to assess the efficacy of HVGs^26^; future studies should consider prioritizing assessments of personal recovery, utilizing subjectively defined outcomes and more thoroughly investigating what keeps individuals returning to HVGs.

The outcomes likewise suggest no changes in the perception of group cohesion or therapeutic factors over the course of the group. This is perhaps surprising, given the emphasis on peer relationships and connections in HVGs,^6,7^ as well as the fact that most participants reported that it was useful to meet other voice hearers and endorsed feeling less alone following group attendance. It is possible that these benefits resulted from learning from others more so than connecting to others (participants did, eg, report learning useful information to make sense of voice hearing but did not report making more friends). This is also potentially an artifact of the groups meeting online where group cohesion and connection are harder to establish.^13^ The precise impact of the online medium on group cohesion will be further extrapolated in the forthcoming qualitative findings.

Several design considerations for future research into HVGs were identified in the present study. In terms of recruitment, we demonstrated the feasibility of recruiting participants across services into a single group. We suggest that future trials endeavor to retain this feature, given that there may be unique benefits of maximizing the diversity of voice hearing experiences within the group.^7^ Furthermore, online groups may represent a promising way of implementing support across services. To maximize ecological validity and remain as true to the values of HVM as possible, this study was open to individuals who were not receiving care from mental health teams. However, while social media referral rates were high, difficulties in establishing the identities and experiences of those recruited online precluded any of these potential participants from being eligible for the study. Given the imperative to ensure the safety of the group members, 2 individuals were excluded following consent but prior to the group commencing as we were unable to verify their identities, voice hearing status, and whether they were motivated to take part in the support group. Future studies within NHS services should thus be mindful of this challenge and potentially opt to only recruit individuals who are currently open to a clinical team. Finally, the tension between the HVM ethos and traditional research methodology must continue to be acknowledged, and future studies should endeavor to promote the values of the HVM within their methodology. For example, while this trial had a prespecified duration (6-month intervention), future studies might instead hold groups over a longer period, have rolling admission to the group, and not prespecify the desired attendance rate (in accordance with the HVM’s practice of enabling participants to engage in groups for as long as feels necessary to them). Similarly, given HVM’s ethos, it may be that a future randomized controlled trial of online HVGs in the NHS is not the most appropriate methodology; pragmatic trials,^49^ patient preference trials^50^ (examining, eg, online HVGs compared to face-to-face HVGs), and trials using subjective goal-based outcomes may be more suitable. Cost-effectiveness should likewise be thoroughly assessed in future work. As an online, peer-delivered intervention, HVGs represent a resource-efficient form of support and may therefore both address some of the resource-related implementation barriers that other forms of psychological interventions face^18^ and provide sufficient support such that participants may need less input from statutory services.^51^

This study must be viewed considering its limitations. We did not include a follow-up assessment after group completion; future studies should include a longer follow-up period to detect the durability of any group effects. Second, as author A.B. also acted as the primary group facilitator, future studies should conduct multiple groups to account for any possible facilitator effects. Finally, most (78%) participants in the current study identified as White; future research would benefit from intentionally trying to recruit a more ethnically diverse sample.

### Conclusion

Overall, the results indicate that it is feasible, acceptable, and safe to conduct an online HVG in the UK NHS, but further research is now needed to evaluate clinical efficacy. As an existing, resource-efficient form of support, further investigation into facilitating online HVGs in the NHS is warranted.
